# Influence of Positive and Threatened Awe on the Attitude Toward Norm Violations

**DOI:** 10.3389/fpsyg.2020.00148

**Published:** 2020-02-19

**Authors:** Kazuki Sawada, Michio Nomura

**Affiliations:** Graduate School of Education, Kyoto University, Kyoto, Japan

**Keywords:** positive awe, threatened awe, norm violation, tolerance, attitude

## Abstract

Awe is an emotional response to vast stimuli needing for accommodation. Although several studies have revealed that awe led to more ethical attitudes toward one’s own behavior and to generosity toward people in general, it is unclear whether and how the two types of awe—positive and threatened—influence one’s attitude toward others’ social norm violations. In the current study, we examined the influence of these types of awe on tolerance toward deviators’ behavior by using a pre-post design and a scenario task within the Japanese population. The findings indicated that positive awe increased the tolerance of others’ norm violations, while threatening awe did not.

## Introduction

Social norms have an important role in maintaining the society and interpersonal relationships in the interaction with ecological and historical threats (e.g., natural disasters and conflicts). Countries and regions which have been exposed to such threats have tight cultures (strong norms and intolerance toward deviators) (Gelfand et al., 2011; Harrington and Gelfand, 2014). Conversely, given that cultural tightness is associated with lower levels of happiness (Harrington et al., 2015) and that disgust for norm violations can sometimes turn into aggression (Bondü and Richter, 2016), the importance of liberalizing attitudes toward norm violations was postulated. Therefore, in this study, we pay attention to awe as the factor of tolerance toward norm violations and investigate this effect in East Asia, specifically Japan, which is thought to be a tight culture (Gelfand et al., 2011; Mrazek et al., 2013).

Awe is an emotional response to stimuli characterized by perceived vastness and need for accommodation (Keltner and Haidt, 2003). Positive awe induces a feeling of being trivial in a vast world, that is “small self” (e.g., Shiota et al., 2007; Piff et al., 2015; Bai et al., 2017; Gordon et al., 2017), decreases aggression (Yang et al., 2016), and facilitates generosity and prosocial behavior toward people in general (Rudd et al., 2012; Piff et al., 2015; Guan et al., 2019; Li et al., 2019). Interestingly, awe-prone participants reported a high score on the openness-to-experience personality trait (Shiota et al., 2006), and high openness is associated with looseness in culture (weak social norms and tolerance toward norm violation) (Harrington and Gelfand, 2014). This implies that positive awe had a receptive effect on norm violation. However, while positive awe experience increased temporally measured tolerance for uncertainty (Valdesolo and Graham, 2014), dispositional awe correlated with the need for closure cognition (Shiota et al., 2006). The relationship between trait-awe and psychological attitude cannot predict the relationship between state-awe and psychological attitude, and little is known about the influence of state-awe on norm violation. Additionally, many previous studies examining the effect of awe on generosity and prosocial tendencies have been conducted within the context of ethical judgment of one’s own selfish behavior and tolerance of people in general (not deviators). Thus, it remains unclear how awe influences attitude toward others’ norm violations in a daily context.

Awe is roughly classified into two types based on valence: positive awe, which is a positive emotion and often induced by the beauty of nature (e.g., the Grand Canyon and aurora) and great people (e.g., Gandhi), and threatened awe, which is awe with a flavor of fear and often induced by the natural disasters (e.g., tsunami and tornado) or notorious dictators such as Adolf Hitler (Gordon et al., 2017). Awe, then, has general and specific effects depending on its valence (Piff et al., 2015; Gordon et al., 2017; Guan et al., 2019). The influence of awe on tolerance toward others’ norm violations may differ depending on the types of awe, as threatened awe does not enhance a sense of connection to everything unlike positive awe (Krenzer, 2018). In line with these findings, the purpose of this paper is to investigate the influence of awe on the attitude toward others’ norm violations using both positive awe and threatened awe.

Positive awe enhances a sense of connection to a particular person (e.g., a friend) or to people in general (Shiota et al., 2007; Van Cappellen and Saroglou, 2012; Krause and Hayward, 2015; Stellar et al., 2017; Nelson-Coffey et al., 2019). In an American sample, positive awe was found to decrease self-focused attention and strengthen the feeling of being included in a community (Bai et al., 2017). Moreover, given that induction of positive awe decreases participants’ social dominance orientation and promotes environmentalism (Zhao et al., 2018), positive awe releases people from their social hierarchy, and connects them not only with one another but also with non-human objects such as the environment. A previous study that has developed the awe state scale has also revealed that “connectedness” and/or “liberation/connection” is an important factor in positive awe-experiences, which includes items such as “connected to everything” (Krenzer, 2018; Yaden et al., 2019). Positive awe liberates people’s attention from the existing self and references, and enhances a sense of connectedness to everything, perhaps depending on the context. Thus, positive awe may promote a tolerant attitude toward social norm violations, in the context of connectedness to deviators. Therefore, we hypothesize that induction of positive awe encourages participants to tolerate norm violation.

While positive awe strengthens a sense of being connected to everything, threatened awe does not (Krenzer, 2018). If positive awe enhances a sense of connection to other people and even deviators, independently of social hierarchy, it is postulated that threatened awe promotes neither a tolerant attitude nor an intolerant attitude toward others’ norm violations; as suggested by previous research, threatened awe may enhance strict attitudes toward norm violations (Gelfand et al., 2011; Gelfand and Lun, 2013; Kastenmüller et al., 2013; Mrazek et al., 2013). Furthermore, cross cultural surveys and experiments indicate that ecological, historical, and societal threats (e.g., natural disaster, terrorism, war) promote tightness and intolerant attitudes toward norm violation (Gelfand et al., 2011; Gelfand and Lun, 2013; Mrazek et al., 2013). Also, the priming of a natural disaster enhances sensitivity to justice against perpetrators (Kastenmüller et al., 2013). However, these studies did not check whether participants felt awe toward the ecological threat nor investigate the effect of threatened awe directly. Considering that natural disasters and the 9.11 attack are typical of stimuli that induce threatened awe (Gordon et al., 2017), threatened awe may encourage participants to be intolerant of norm violations.

Positive awe may be related to the socio-political factors in the tightness-looseness model (e.g., government, education, religion and spirituality), because awe is often induced by great leaders, scientific knowledge, and religion (e.g., Keltner and Haidt, 2003; Gordon et al., 2017; Valdesolo et al., 2017). Previous studies have proposed a systems model of tightness-looseness and investigated the factors of the cultural tightness-looseness from the perspective of temporal behavior and culture (e.g., Gelfand et al., 2011; Gelfand and Lun, 2013), but most of them have focused on the promotors of tightness, and the socio-political factor is one of them. In this study, we examine the temporal influence of positive awe as the promotor of looseness.

### Current Study

The purpose of this study is to examine whether and how two types of awe (positive awe/threatened awe) influence attitudes toward others’ norm violations. As norms differ across countries, regions, and societies, it may be difficult to generalize the study. Therefore, we measure attitudes toward norm violations by using the social norm violation scenario task (Mu et al., 2015), which comprehensively depicts norm violations in various situations and has been validated between tight and loose cultures. We tested the hypothesis that positive awe encourages participants to decrease the ratings of “inappropriateness” in norm violation scenarios, while threatened awe does not, or not otherwise encourages intolerance of norm violations.

## Materials and Methods

### Participants

The participants of this study comprised 50 Japanese-speaking students from Kyoto University (22 women; mean age = 21.32, *SD* = 2.04). This study was approved by the Ethical Review Board of Kyoto University. All participants provided written informed consent. For ethical considerations, it was explained that they might watch a video depicting natural disasters and had the right to exit the experiment at any time. We also checked whether the ratings of each of the measures were reliable, based on the criterion of Mean ± 3 *SD*. As a result, two participants were excluded from analysis. Finally, 48 participants (20 women; mean age = 21.29, *SD* = 2.04) in the final sample were analyzed.

### Procedures

First, all participants watched a 2 min neutral clip, in which a narrator described an automobile factory. Participants were inside an experiment room. The videos were presented on computer and it was listened to by speakers. After watching a neutral video, participants completed measures of their emotional states, perceived self-size (Bai et al., 2017), sense of connection with their community (Mashek et al., 2007), attitude toward social norm violations (Mu et al., 2015), openness-to-experience (Costa and McCrae, 1992), conscientiousness (Costa and McCrae, 1992) and other scales (pretest). Then, they were randomly assigned to watch one of two videos: a 2 min positive awe-eliciting clip, consisting of a montage of beautiful nature clips, composed of glaciers, forests, mountains, and stars; a 2 min threatened awe-eliciting clip, consisting of a montage of threat-based nature clips, specifically tsunamis and floods. After watching an awe video, they completed the same measures of their emotional states, perceived self-size, sense of connectedness with their community, attitude toward social norm violation, openness-to-experience, conscientiousness and other scales (posttest). After this experiment, participants in a threatened awe condition were asked whether they or their family had experienced a natural disaster, and none reported having had such an experience. The neutral and positive awe-eliciting clips used in this study were the same clips used in a previous study (Takano and Nomura, 2018). We used this threatened awe-eliciting clips without measuring its validity by preparatory experiment, but ratings of “ifu,” “ike,” and “perceived self-size” provided us with the validity of this video and manipulation. Participants responded to emotion, perceived self-size, sense of connectedness with their community in writing and the rest using Qualtrics^1^. Screenshots of each conditions were shown in Supplementary Figure S1.

### Materials

#### Emotions

As a manipulation check, we used these emotion reports to confirm that participants felt awe while watching the awe-inducing video more so than while watching a neutral video. All participants reported the extent to which they were feeling: “ike,” “ifu” (awe is referred to as “*ike*” and/or “*ifu*” in Japan; Muto, 2014), wonder, fear, anxiety, amazement, annoyance, compassion, moved, nervous, respect, sadness, curious, amusement, happiness, and appreciation, measured on a 7-point Likert scale from 1(*not at all*) to 7(*extremely*). This measure is widely used to check the validity of awe induction (see Gordon et al., 2017).

#### Perceived Self-Size

Perceived self-size was measured with one item, symbolic self-circle, taken from Bai et al. (2017). Participants checked the circle that best represented how big or small they feel themselves to be on a 7-point Likert scale rating from 1 (*a smallest circle*) to 7 (*a biggest circle*). This scale consists of one pictorial item and is insusceptible to translation issue. Also, it has been validated across cultures (Bai et al., 2017).

#### A Sense of Connection With the Community and Society

In the same way as a previous study (Bai et al., 2017), we measured participants’ sense of connection with their community and society using the Inclusion of Community in the Self scale (Mashek et al., 2007), a single-item pictorial measure consisting of six pairs of overlapping circles, with each pair of same-sized circles overlapping slightly more than the preceding pair. In each pair, the left circle was labeled as “self” and the right as “community at large.” Participants checked the pair of circles that best represented their relationship with their community on a 6-point Likert scale from 1 (*not at all overlapping*) to 6 (*mostly overlapping*).

#### Openness to Experience and Conscientiousness

We measured participants’ openness to experience and conscientiousness at the state level to examine the pre-post effects of presentation of positive and threatened awe for each, using the Japanese version (Shimonaka et al., 1999) of the NEO-FFI (Costa and McCrae, 1992). The NEO-FFI-Openness to Experience (α = 0.58) and NEP-FFI-Conscientiousness (α = 0.78) consisted of 12 items, respectively. Participants were asked to rate the extent to which they agreed with each statement, from 0 (strongly disagree) to 4 (strongly agree) to twenty-four items.

#### Attitude Toward Norm Violation

We measured participants’ attitudes toward others’ social norm violations by using a scenario task, the Social Norm Violation Task (Mu et al., 2015). This task has been validated across cultures in a previous study, controlling for social norm differences between cultures. Participants were asked to judge whether certain behaviors were appropriate or not in different situations. Subjects were presented with forty-five scenarios (fifteen scenarios × three conditions): fifteen “appropriate” scenarios each describing a stranger behaving appropriately in a situation (e.g., Jacob is in the bike lane. He is cycling.); fifteen “weak violation” scenarios each describing a stranger behaving weakly inappropriately in a situation (e.g., Jacob is on the city sidewalk. He is cycling.); fifteen “strong violation” scenarios each describing a stranger behaving strongly inappropriately in a situation (e.g., Jacob is on the highway. He is cycling.). Participants were asked to judge the level of inappropriateness for all scenarios on a 6-point Likert scale from 1 (very appropriate) to 6 (very inappropriate). In previous studies (Mu et al., 2015), participants were asked to rate appropriateness from 1 (*very appropriate*) to 4 (*very inappropriate*). We added two points (“*appropriate*” and “*inappropriate*”) to the four points (“*very appropriate*,” “*slightly appropriate*,” “*slightly inappropriate*,” and “*very appropriate*”) used in the previous study (Mu et al., 2015) to analyze the ratings serially and quantitatively. We translated this tool into Japanese and used it. The mean ratings of “inappropriateness” for each condition in fifteen scenarios in each condition were analyzed as dependent variables.

## Results

### Emotion (Manipulation Check)

Emotion reports confirmed that participants in a positive awe condition experienced stronger feelings of both of “*ike*” and “*ifu*” in the posttest (i.e., after watching positive awe clips) (“*ike*”: *M* = 4.92, *SD* = 1.86; “*ifu*”: *M* = 4.25, *SD* = 1.77) than in the pretest (i.e., after watching neutral clips) (“*ike*”: *M* = 2.42, *SD* = 1.42; “*ifu*”: *M* = 1.38, *SD* = 0.64) [“*ike*”: *F*(1,23) = 35.20, *p* < 0.001, $\eta_{p}^{2}$ = 0.61; “*ifu*”: *F*(1,23) = 68.42, *p* < 0.001, $\eta_{p}^{2}$ = 0.75] (see Table 1). Participants in threatened awe conditions experienced stronger feelings of both of “*ifu*” and “*ike*” in the posttest (i.e., after watching threatened awe clips) (“*ifu*”: *M* = 5.54, *SD* = 1.25; “*ike*”: *M* = 3.75, *SD* = 2.17) than in the pretest (“*ifu*”: *M* = 1.17, *SD* = 0.48; “*ike*”: *M* = 1.75, *SD* = 1.36), [“*ifu*”: *F*(1,23) = 296.69, *p* < 0.001, $\eta_{p}^{2}$ = 0.93; “*ike*”: *F*(1,23) = 13.80, *p* < 0.001, $\eta_{p}^{2}$ = 0.38] (see Table 1). Participants in threatened awe conditions experienced stronger feelings of “*ifu*” than did participants in positive awe conditions in the posttest minus pretest, *F*(1,46) = 12.51, *p* < 0.001, $\eta_{p}^{2}$ = 0.21. The difference in “*ike*” rating (posttest minus pretest) between positive awe condition and threatened awe condition was not significant, *F*(1,46) = 0.54, $\eta_{p}^{2}$ = 0.01. These analyses were performed by an ANOVA. Other emotional state reports were shown in Supplementary Table S1.

**TABLE 1:** Mean differences in awe emotion, perceived self-size, A sense of connecting with community and society, attitude toward norm violation across conditions.

	**Positive awe(*N* = 24)**				**Threatened awe(*N* = 24)**				**Comparison of two types of awe (posttest-pretest)**	
							
**Variable**	**Pre**	**Post**	***F***	**$\eta_{p}^{2}$**	**Pre**	**Post**	***F***	**$\eta_{p}^{2}$**	**Interaction *F***	**$\eta_{p}^{2}$**
**Awe emotion**										
Ifu	1.38 (0.64)	4.25 (1.77)	68.42**	0.75	1.17 (0.48)	5.54 (1.25)	296.69**	0.93	12.15	0.21
Ike	2.42 (1.42)	4.92 (1.86)	35.20**	0.61	1.75 (1.36)	3.75 (2.17)	13.80**	0.38	0.54	0.01
**Perceived self-size**										
Symbolic self-circle	3.96 (1.23)	3.42 (1.61)	6.76*	0.23	4.00 (1.29)	3.21 (1.47)	7.24**	0.24	0.48	0.01
**Attitude for norm violation**										
Appropriate	1.73 (0.36)	1.72 (0.41)	0.02	0.00	1.67 (0.41)	1.72 (0.54)	1.05	0.04	0.64	0.01
Weak violation	4.02 (0.50)	4.00 (0.48)	0.50	0.02	3.98 (0.50)	4.03 (0.52)	1.14	0.05	1.65	0.03
Strong violation	4.95 (0.33)	4.83 (0.38)	6.92*	0.23	4.94 (0.45)	4.94 (0.48)	0.02	0.00	3.25^+^	0.07

Each mean is followed by the corresponding SD in parentheses. Awe is called “ifu” or “ike” in Japan. ^+^*P* < 0.10; *P < 0.05; **P < 0.01.

### Perceived Self-Size

Perceived self-size reports confirmed that participants in positive awe conditions perceived smaller self-sizes in the posttest (*M* = 3.42, *SD* = 1.61) than in the pretest (*M* = 3.96, *SD* = 1.23), *F*(1,23) = 6.76, *p* = 0.016, $\eta_{p}^{2}$ = 0.23. Participants in threatened awe conditions perceived smaller self-sizes in the posttest (M = 3.21, *SD* = 1.47) than in the pretest (*M* = 4.00, *SD* = 1.29), *F*(1,23) = 7.24, *p* = 0.013, $\eta_{p}^{2}$ = 0.24. These analyses were performed by an ANOVA. Accordingly, for both awe conditions, ratings of “*ifu*” and “*ike*” in the posttest increased compared to the pretest, and ratings of “perceived self-size” in the posttest decreased from the pretest.

### Openness to Experience and Conscientiousness

Ratings for openness to experience and conscientiousness did not differ between pretest and posttest for either awe condition, *Fs* < 0.66, $\eta_{p}^{2}$s < 0.03 (see Supplementary Table S2).

### A Sense of Connection With Community and Society

Ratings for inclusion of community did not differ between pretest and posttest for either awe condition, *Fs* < 0.52, $\eta_{p}^{2}$*s* < 0.02 (see Supplementary Table S2).

### Attitude Toward Norm Violation

**FIGURE 1:**
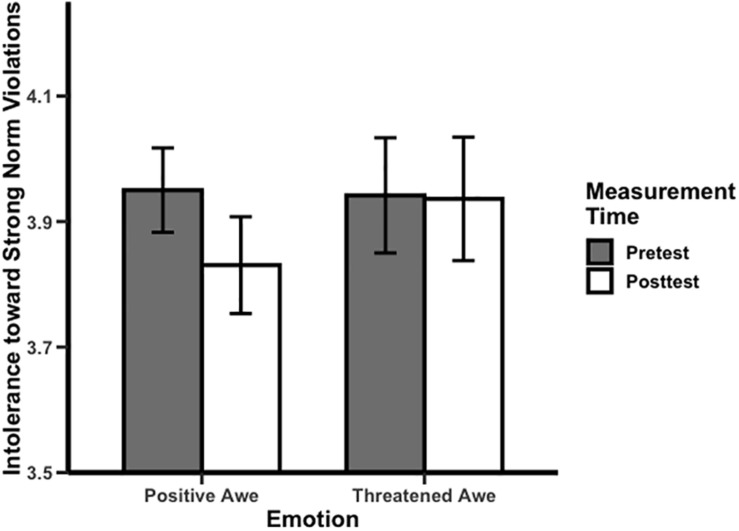
Influences of positive awe and threatened awe on the intolerance toward strong norm violations. Error bars represent ±1 SE.

Testing our hypothesis that positive awe encourages tolerance toward norm violations, we performed an ANOVA on the ratings of inappropriateness for scenarios depicting strong violations, which revealed the predicted positive awe effect, *F*(1,23) = 6.92, *p* = 0.015, $\eta_{p}^{2}$ = 0.23. Participants in positive awe conditions reported the more tolerant attitudes toward strong violations in the posttest (*M* = 4.83, *SD* = 0.38) than in pretest (*M* = 4.95, *SD* = 0.33). Ratings for appropriate and weak violation scenarios in positive awe conditions did not differ between pretest and posttest, *Fs* < 0.50. In turn, to test whether inducing threatened awe influenced attitudes toward norm violations, an ANOVA on the rating of for inappropriateness for scenarios depicting strong violations revealed an insignificant effect, *F*(1,23) = 0.02, $\eta_{p}^{2}$ = 0.00. This result supported the hypothesis that threatened awe did not encourage a tolerant attitude toward violators, unlike positive awe. Ratings of appropriate and weak violation scenarios in threatened awe conditions did not differ between pretest and posttest, *Fs* < 1.14, $\eta_{p}^{2}$*s* < 0.05. Moreover, we performed an ANOVA on the changed rating (posttest minus pretest) of inappropriateness for strong violation scenarios, which revealed marginally significant condition effects, *F*(1,46) = 3.25, *p* = 0.078, $\eta_{p}^{2}$ = 0.07, 95%CI [0.000,0.230] (see Figure 1). This result remained when controlling the changed rating of openness to experience and conscientiousness as covariates, *F*(1,44) = 3.27, *p* = 0.084, $\eta_{p}^{2}$ = 0.078, 95%CI [0.000,0.231].

## Discussion

The purpose of this study was to examine the influence of two types of awe (positive awe and threatened awe) on attitudes toward others’ norm violations. To the best of our knowledge, this study is the first that shows different effects of two types of awe on tolerant attitudes toward others’ norm violation: positive awe encouraged people to tolerate others’ strong norm violations, but threatened awe did not.

### Positive Awe and Attitude Toward Norm Violation

Induction of both positive and threatened awe increased ratings of awe (“*ifu*” and “*ike*”), and decreased the rating of “perceived self-size.” Manipulation checks allowed for the validation of awe induction based on the experimental stimulus. Additionally, the rating of “inappropriate” for strong norm violations decreased after viewing positive awe clips. This result corresponds with previous studies that found that positive awe enhances tolerance of people in general by using the money donation task, helping time task, and dictator game (Rudd et al., 2012; Piff et al., 2015; Guan et al., 2019).

On the other hand, interestingly, positive awe did not significantly change the Inclusion of Community in the Self (a measurement of a sense of belonging to society and community). As effects of positive awe on the social networks vary cultually (Bai et al., 2017), the present finding differs from that of the various ethnic samples recruited through Amazon MTurk in the United States (Bai et al., 2017). However, it corresponds with the findings within Chinese population, showing that positive awe did not significantly change the sense of social network size among Chinese people (Bai et al., 2017), who live in a collectivist culture like the Japanese. It is also noteworthy that positive awe led to a closer distance between the self and others in the Chinese sample (Bai et al., 2017). Given that a closer connection to society and community leads to more intolerance of others disturbing order, as typical of collectivism and tightness of culture (Gelfand et al., 2011), the present result in which attitudes toward others’ norm violations become tolerant may be consistent with the result that a feeling of connection with society and community is not changeable.

Our findings also contribute toward furthering the theory in the domain. Previous research has mainly focused on the factors of tightness, but this study focused on positive awe as a promotor of looseness and found that positive awe encouraged participants to hold loose attitudes. Further, positive awe is induced mainly by nature, another person, self, religious experiences, art, music, and architecture (Gordon et al., 2017). Although, according to previous studies, religiosity is associated with tightness (Gelfand et al., 2011), our results suggest that certain religions (e.g., a religion which has a loving God) are associated with looseness. We may need to consider how people perceive God within religion, in the tightness-looseness model. This argument corresponds recent findings that conflicts increase support for cultural tightness, which in turn increases the importance of punitive God (Caluori et al., 2020).

### Difference Between the Two Types of Awe

Participants in a threatened awe condition reported higher changed rating of “*ifu*” than participants in a positive awe condition, while in regard to changed rating of “*ike*,” there was no significant difference. This may be because the letter “
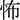
” in “*ifu* (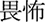)” means fear in Japanese, while the letter “
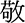
” in “*ike* (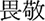)” means respect.

In contrast to positive awe, threatened awe did not have a significant influence on the attitudes toward others’ norm violations. The interaction, although it was marginally significant, indicated that positive awe and threatened awe have, at least, a different influence on the generosity at the least. Furthermore, the present result corresponds to the previous hypothesis that effects on connection would differ between the types of awe (e.g., eliciter, valence) (Van Cappellen and Saroglou, 2012; Krenzer, 2018), suggesting that positive awe may promote the generosity toward norm violation by emphasizing connection to others with various attributes, while threatened awe does not, because it does not emphasize connection. On the other hand, threatened awe often leads to the enhancement of connections between people and other-focused behavior and culture, alleviating feelings of loss in the face of threatened-awe inducing events (Nomura et al., in press). Moreover, as norms have an important role in maintaining the society, tolerance toward deviators may not be adaptive in such a situation. In sum, positive awe enhances connectedness to everything/everyone, whereas threatened awe may lead to connection with a particular something/someone, which does not include deviators, probably because connection to deviators generally does not become a buffer against threats. This explanation corresponds to the result that threatened awe encourages participants to be generous toward people in general (Piff et al., 2015). Since few studies have examined which layer of “connectedness” is affected by experience of awe, investigation of the influence of two types of awe on the feeling of connectedness is of interest.

## Limitations and Future Directions

While our study contributes to furthering the theory in understanding attitudes toward norm violation, certain limitations of the current study should be noted, along with some future directions. First, the sample size is relatively small for a behavioral study. This may have contributed to the marginally significant condition effects (positive awe vs. threatened awe) observed in our study.

Second, as the influence of awe on the attitudes toward norm violation may vary between cultures, it is of interest to see whether the effect of positive awe on generosity toward norm violations is reproduced in Western samples.

Third, details of the psychological processes underlying enhancement of tolerance toward norm violations by inducing positive awe remains unclear. Investigations into the nature and effect of awe on individuals and society are in nascent stages and researchers have a great deal yet to explore; for example, the effect of elicitor of awe on social cognition and time perception. Time perception has also been mentioned as an effect that differs between positive and threatened awe (Guan et al., 2019). Social norm violation is disorderly behavior, and ethnical judgment of such behavior may tolerate it in states liberated from time pressure.

Fourth, in this study, a neutral video was shown before positive or threatened awe video in a fixed order, so it is possible to interpret the results as participants being primed to experience something more interesting in general after watching the neutral clips. However, considering awe experiences themselves consist of interesting and wonderous things (e.g., Gordon et al., 2017), it is likely that the present results revealed awe-specific effects under the comparison of two types of awe. It is also of interest to see whether neutral states preceding awe experience would affect attitude toward norm violations.

The forms of connections induced by awe should be investigated in future studies as a function of collectivist vs. individualistic cultures that the participants belong to, as well as within culture differences examining how individuals view their own cultures. Since there is little awe-related research to examine the forms and layers of connection (except for Van Cappellen and Saroglou, 2012), we investigated the influence of awe on general connectedness from the perspective of norm violation. In this study, however, the results for the Inclusion of Community in the Self scale were not significant. This may be because the meaning of “community” in the ICS varies between participants, and some of them might have friends in mind while other may think about family members. Different measures than the ICS may be more appropriate for collectivist cultures. Also, as religiosity and spirituality regulated the influence of awe on feelings of connectedness, religiosity may be related to the cultural differences in the influence of awe on connection (Nomura et al., in press).

Finally, the homogeneity of the sample population may be considered a weakness, particularly since students’ data may not generalize to the wider population. Future studies should explore additional populations to improve our understanding of this phenomenon.

## Conclusion

In summary, we investigated the influence of two types of awe on attitude toward others’ norm violations. The results indicated that positive awe led to tolerant attitudes toward others’ norm violations, while threatened awe did not, suggesting that the influence of awe on attitude toward others’ norm violations differs with the types of awe.

## Data Availability Statement

The datasets generated for this study are available on request to the corresponding author.

## Ethics Statement

The studies involving human participants were reviewed and approved by the Ethical Review Board of Kyoto University. The patients/participants provided their written informed consent to participate in this study.

## Author Contributions

Both authors designed the current study, analyzed the date, interpreted the funding and reviewed the manuscript. KS recruited the participants, collected the data and wrote the main manuscript.

## Conflict of Interest

The authors declare that the research was conducted in the absence of any commercial or financial relationships that could be construed as a potential conflict of interest.

## Footnotes

1 https://www.qualtrics.com/jp/
