# Supplementary material for: Influence of Positive and Threatened Awe on the Attitude Toward Norm Violations
Source: Front Psychol. 2020 Feb 19;11:148. doi: 10.3389/fpsyg.2020.00148 (PMC7042396; doi:10.3389/fpsyg.2020.00148)
Supplement: Supplementary file 1 [file Data_Sheet_1.docx]

| **Supplementary Table 1** | | | | | | | | | | | | | | | | | | | | | |  |
| --- | --- | --- | --- | --- | --- | --- | --- | --- | --- | --- | --- | --- | --- | --- | --- | --- | --- | --- | --- | --- | --- | --- |
| **Mean Differences in Emotion Across Conditions** | | | | | | | | | | | | | | | | | | | | | |  |
|  |  |  | Positive awe (*N* = 24) | | |  |  |  |  |  | Threatened awe (*N* = 24) | | |  |  |  |  |  |  |  |  | |
| Variable | |  | Pre |  | Post |  | *F* |  | $\eta^{2}$ |  | Pre |  | Post |  | *F* |  | $\eta^{2}$ |  | Interaction *F* |  | $\eta^{2}$ | |
| Ike | |  | 2.42 (1.42) |  | 4.92 (1.86) |  | 35.20 | *** | .61 |  | 1.75 (1.36) |  | 3.75 (2.17) |  | 13.80 | ** | .38 |  | 0.54 |  | .01 | |
| Ifu | |  | 1.38 (0.64) |  | 4.25 (1.77) |  | 68.42 | *** | .75 |  | 1.17 (0.48) |  | 5.54 (1.25) |  | 297.68 | *** | .93 |  | 12.15 | ** | .21 | |
| Wonder | |  | 2.25 (1.36) |  | 4.58 (1.79) |  | 47.45 | *** | .67 |  | 1.92 (1.50) |  | 5.79 (1.18) |  | 108.17 | *** | .83 |  | 9.37 | ** | .17 | |
| Fear | |  | 1.29 (0.69) |  | 3.17 (1.79) |  | 25.33 | *** | .52 |  | 1.21 (0.66) |  | 6.13 (0.85) |  | 559.88 | *** | .96 |  | 50.84 | ** | .53 | |
| Anxiety | |  | 1.71 (1.36) |  | 2.33 (1.40) |  | 3.14 | ＋ | .12 |  | 1.46 (1.06) |  | 5.04 (1.76) |  | 86.61 | *** | .79 |  | 35.07 | ** | .43 | |
| Amazement | |  | 3.50 (1.75) |  | 4.58 (1.44) |  | 9.55 | ** | .29 |  | 2.83 (2.01) |  | 4.54 (1.74) |  | 10.53 | ** | .31 |  | 0.98 |  | .02 | |
| Annoyance | |  | 2.63 (1.66) |  | 1.88 (1.36) |  | 5.13 | * | .18 |  | 1.67 (1.44) |  | 3.79 (2.04) |  | 15.92 | ** | .41 |  | 21.01 | ** | .31 | |
| Compassion | |  | 1.54 (1.06) |  | 1.38 (0.71) |  | 0.49 |  | .02 |  | 1.21 (0.66) |  | 4.42 (1.86) |  | 72.89 | *** | .76 |  | 57.53 | ** | .57 | |
| Moved | |  | 2.75 (1.51) |  | 5.04 (1.85) |  | 28.16 | *** | .55 |  | 2.50 (1.62) |  | 2.33 (1.81) |  | 0.13 |  | .01 |  | 15.14 | ** | .25 | |
| Nervous | |  | 2.79 (1.77) |  | 1.71 (1.27) |  | 9.84 | ** | .30 |  | 1.46 (0.98) |  | 2.25 (1.78) |  | 3.52 | ＋ | .13 |  | 11.85 | ** | .21 | |
| Respect | |  | 3.21 (1.59) |  | 3.75 (2.13) |  | 1.07 |  | .04 |  | 2.75 (1.87) |  | 1.54 (1.06) |  | 8.96 | ** | .28 |  | 6.99 | ** | .13 | |
| Sadness | |  | 1.29 (0.91) |  | 1.79 (1.14) |  | 3.14 | ＋ | .12 |  | 1.21 (0.66) |  | 4.71 (2.03) |  | 73.50 | *** | .76 |  | 36.53 | ** | .44 | |
| Curious | |  | 3.96 (1.55) |  | 5.46 (1.38) |  | 27.00 | *** | .54 |  | 3.79 (1.56) |  | 3.00 (2.45) |  | 2.22 |  | .09 |  | 14.35 | ** | .24 | |
| Amusement | |  | 3.46 (1.32) |  | 4.17 (1.86) |  | 4.54 | * | .17 |  | 3.00 (1.62) |  | 1.17 (0.48) |  | 32.36 | ** | .59 |  | 30.15 | ** | .39 | |
| Happiness | |  | 2.54 (1.35) |  | 3.75 (1.65) |  | 13.44 | ** | .37 |  | 2.50 (1.56) |  | 1.25 (0.85) |  | 16.43 | ** | .42 |  | 29.66 | ** | .39 | |
| Appreciation | |  | 2.71 (1.71) |  | 1.92 (1.14) |  | 5.97 | * | .21 |  | 1.96 (1.33) |  | 1.96 (1.57) |  | 0.00 |  | .00 |  | 2.93 |  | .06 | |
| *Note.* Each mean is followed by the corresponding *SD* in parentheses. All emotional state reports were measured on a 7-point Likert scale from 1 (*not at all*) to 7 (*extremely*). | | | | | | | | | | | | | | | | | | | | | |  |
|  | Awe is called "ifu" or "ike" in japan. | | | | | | | | | | | | | | | | | | | | |  |
|  | ＋*p*< 0.10; **p* < 0.05; ***p* < 0.01; ****p* < 0.001. | | | | | | | | | | | | | | | | | | | | |  |

| **Supplementary Table 2** | | | | | | | | | | | | | | | | | | | | | | |
| --- | --- | --- | --- | --- | --- | --- | --- | --- | --- | --- | --- | --- | --- | --- | --- | --- | --- | --- | --- | --- | --- | --- |
| **Mean Differences in Openness to Experience, Conscientiousness, and Collective Orientation Across Conditions** | | | | | | | | | | | | | | | | | | | | | | |
|  | |  |  | Positive awe (*N* = 24) | | |  |  |  |  |  | Threatened awe (*N* = 24) | | |  |  |  |  |  |  |  |  |
| Variable | | |  | Pre |  | Post |  | *F* |  | $\eta^{2}$ |  | Pre |  | Post |  | *F* |  | $\eta^{2}$ |  | Interaction *F* |  | $\eta^{2}$ |
| Openness to Experience | | |  | 2.74 (0.42) |  | 2.75 (0.42) |  | 0.14 |  | .01 |  | 2.39 (0.48) |  | 2.39 (0.47) |  | 0.00 |  | .00 |  | 0.06 |  | .00 |
| Conscientiousness | | |  | 2.22 (0.68) |  | 2.22 (0.71) |  | 0.00 |  | .00 |  | 2.24 (0.58) |  | 2.22 (0.59) |  | 0.66 |  | .03 |  | 0.18 |  | .00 |
| Collective Orientation | | |  | 3.71 (1.16) |  | 3.58 (1.10) |  | 0.18 |  | .01 |  | 3.67 (1.31) |  | 3.54 (1.47) |  | 0.52 |  | .02 |  | 0.00 |  | .00 |
| *Note.* Each mean is followed by the corresponding SD in parentheses. Openness to Experience and Conscientiousness were measured on a 5-point Likert scale from 0 (*strongly disagree*) to 4 (*strongly agree*) by the Japanese version (Shimonaka et al., 1999) of NEO-FFI (Costa & McCrae, 1992). Collective orientation was measured on a 6-point Likert scale (1 = *not at all overlapping to* 6 = *mostly overlapping*) by the Inclusion of Community in the Self Scale (Mashek et al., 2007). | | | | | | | | | | | | | | | | | | | | | | |
|  | ＋*p* < 0.10; **p* < 0.05; ***p* < 0.01; ****p* < 0.001. | | | | | | | | | | | | | | | | | | | | | |


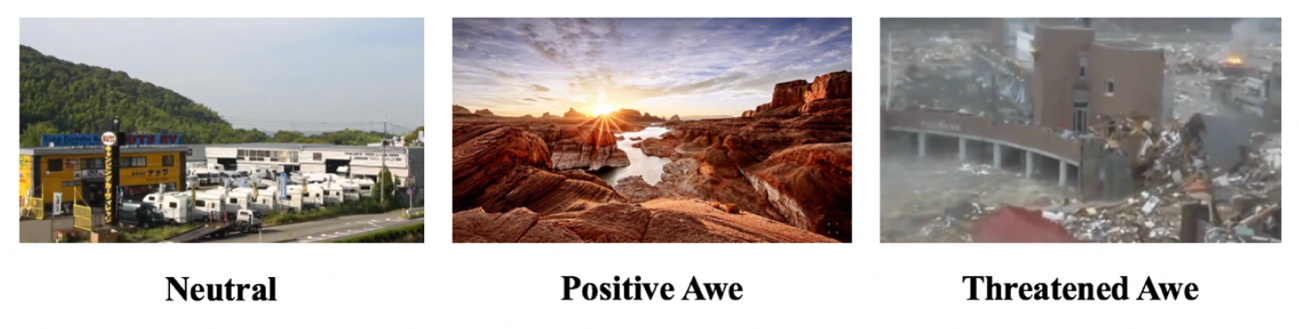


**Supplementary Figure 1.**

These pictures are screenshots of each condition (neutral, positive awe, and threatened awe). In neutral clips, a narrator described an automobile factory. Positive awe clips consisted of a montage of beautiful nature clips, composed of glaciers, forests, mountains, and stars. Threatened awe clips consisted of a montage of threat-based nature clips, specifically tsunami and floods, because these disasters were more familiar to the Japanese than disasters such as volcano and thunderstorms were. The neutral and positive awe videos were the same as used in aa previous study (Takano & Nomura, 2018).

**Reference.**

Costa, P. T., & McCrae, R. R. (1992). *Revised NEO Personality Inventory (NEO-PIR) and NEO Five Factor Inventory (NEO-FFI) professional manual.* Odessa, FL: Psychological Assessment Resources.

Mashek, D., Cannaday, L. W., & Tangney, J. P. （2007）. Inclusion of community in self scale: A single-item pictorial measure of community connectedness. *Journal of Community Psychology, 35,* 257–275.

Shimonaka, Y., Nakazato, K., Gondo, Y., & Takayama, M. (1999). *Revised NEO-Personality Inventory (NEO-PI-R) and NEO Five- Factor Inventory (NEO-FFI) manual for the Japanese version.* Tokyo: Tokyo Shinri.

Takano, R. & Nomura, M. （2018）. The dark side of awe: From the perspective of intergroup conflict. *The 19th Annual Meeting of Society for Personality and Social Psychology*, Atlanta, Georgia, USA, March 1-3.
